# The STOPBANG score is effective for obstructive sleep apnea syndrome screening and correlates with its features, in a sub-Saharan African population

**DOI:** 10.11604/pamj.2020.36.93.17805

**Published:** 2020-06-15

**Authors:** Massongo Massongo, Adamou Dodo Balkissou, Corine Kenne Kenyo, Brice Nouga Sawa, Nadine Kanko, Eric Walter Pefura

**Affiliations:** 1 Pulmonology Service, Jamot Hospital Yaoundé, Yaoundé, Cameroon; 2 Higher Institute of Medical Technology of Nkolondom, Yaoundé, Cameroon; 3 Faculty of Medicine and Biomedical Sciences, University of Yaoundé 1, Yaoundé, Cameroon; 4 Faculty of Health Sciences, Abdou Moumouni University of Niamey, Niamey, Niger

**Keywords:** Sleep apnea syndrome, diagnosis, STOPBANG, Cameroon, sub-Saharan Africa

## Abstract

**Introduction:**

the STOPBANG score is an easy-to-use screening tool for obstructive sleep apnea (OSA), which has not been validated in sub-Saharan Africa (SSA). We sought to evaluate its diagnostic performance in Cameroun.

**Methods:**

this community-based study took place in a sub-urban area, from November 2015 to April 2016. Adults aged ≥19 years underwent a clinical assessment, including the STOPBANG and the Epworth sleepiness scale (ESS) questionnaires. A respiratory polygraph (RP) was performed on a randomly selected sample. Diagnosis performance included sensitivity (Se), specificity (Sp), and positive and negative predictive values (PPV and NPV). An association was sought between STOPBANG and OSA features.

**Results:**

a total of 3033 were interviewed, of whom 102 had a RP. Their mean age was 49.1±17.9 years, the sex ratio was 1 and the mean body mass index 29.1±6.1 kg/m^2^. For OSA screening (apnea-hypopnea index (AHI) ≥5), the STOPBANG score at threshold 3 obtained: Se=82.9%, Sp=34.4%, PPV=45.9% and NPV=75.0%. For moderate-to-severe OSA (IAH ≥15), these values were 93.3%, 31.1%, 18.9% and 96.4% respectively. Furthermore, STOPBANG-based high risk of OSA correlated with AHI (9.1±10.7/hr vs 3.8±3.5/hr, p=0.0003) and oxygen desaturation index (6.4±7.9/hr vs 2.6±2.1/hr, p=0.0004). There was a non-significant association with ESS (6.3±5.3 vs 4.5±3.5, p=0.06).

**Conclusion:**

in this Cameroonian population, the STOPBANG diagnostic performance did not differ from the original Caucasian one. It could therefore be proposed on a larger scale, since obesity and other OSA risk factors are increasing in SSA.

## Introduction

Obstructive sleep apnea-hypopnea syndrome (OSAHS) is frequent in the adult population. Its prevalence ranged in historical Western and Asian studies between 3 to 7% and 1 to 3% for men and women respectively [1-4]. However, a recent global review showed that nearly one billion (936 million) of adults aged 30-69 years have mild to severe OSAHS, based on an AHI cut off of five or more [5]. The authors used a newly developed algorithm to estimate OSAHS prevalence in countries without prevalence data, by matching them to similar countries, on the basis of body mass index, race and geographical proximity. They found a wide range for OSAHS prevalence: from 7.9% (Macao) to 77.2% (Bruneï and Malaysia). The Cameroon prevalence was estimated 36.7%, and those of the surrounding countries ranged from 18.5% (Central African Republic) to 60.2% (Nigeria) [5]. OSAHS has been associated to higher incidence of high blood pressure, stroke, heart failure, cardiac arrhythmias and coronary artery disease [6-12]. The risk of road accidents related to excessive sleepiness is also well known among truck drivers [13, 14], as well as cognitive and social impairment and a significant increase in mortality [6, 15-17]. These features lead to consider OSAHS as public health problem, which mobilizes adequate health care measures in the Western world.

The optimal diagnosis of OSAHS is easy and reliable, but requires devices that are not uniformly affordable, especially in the sub-Saharan African (SSA) context, leading to under diagnosis and difficulties to assess the real burden of this condition. Besides this limitation, we can assume that there is an epidemiological increase in OSAHS incidence and prevalence in SSA, consistent with the global expansion of chronic non-communicable diseases (NCD). Cardiovascular diseases, diabetes and obesity increased their prevalence over the last 20 years [18-26], and it is estimated that 80% of NCDs-related mortality will come from SSA in the next 5 years [18]. OSAHS is frequently associated with these NCDs as an independent cardiovascular risk factor, but also as comorbidity for diabetes and obesity. Therefore, it is important for caregivers in SSA to have effective diagnosis tools, or otherwise appropriate screening tools in these resource-limited settings. The STOPBANG questionnaire is a validated and reliable screening tool for OSAHS. It was developed in Canadian pre-operative patients waiting for elective surgery [27, 28], and then used on a large scale. However, to our knowledge, its diagnostic performance has not been assessed in SSA. The present study aimed to estimate this performance and other features among general population in Cameroon, Central Africa. It was nested in a wider respiratory health epidemiological study led in the West Region of Cameroon.

## Methods

**Setting and period:** this cross-sectional study took place in Bandjoun, a sub-urban area and headquarters of the Koung-Khi division, West Region, Cameroon. With a 353 km^2^ area and 119,251 inhabitants, Bandjoun had 337 inhabitants/km^2^ in 2015. The study was conducted over a 6-months period, from November 2015 to April 2016. Adults aged ≥19 years living in the study area and with no cognitive nor sensory impairment were invited to participate.

**Sampling:** for the overall study sample, a 2-levels clusters sampling was performed. At the first level, we randomly selected 6 health areas out of the 13 in Bandjoun health district. At the second level, a given number of households were randomly selected in each health area, with a sampling rate ranging from 3 to 14, depending on the population density. Then, all eligible subjects in each selected household were invited to participate in the survey. For the nocturnal respiratory polygraph (RP) group composition, we applied an additional random sampling among the overall sample, so that 20% of participants with high risk of OSAHS (HR-OSAHS) and 3% of those with low risk of OSAHS were selected.

**Data collection and measurements:** seventh year trained medical students collected data, during a physical interview with each participant and using a comprehensive computerized questionnaire. Each subject or one of his relatives reported socio-demographic data, medical history, and symptoms. A physical examination was performed to collect anthropometric data and any other abnormality. We measured the height using a wooden rod of local manufacture, and the weight using an electronic weighing scale. Body mass index (BMI) was subsequently calculated using the formula Weight/ (Height)^2^. The blood pressure was given by an electronic sphygmomanometer, and other measurements (neck and waist circumference) by a usual tape. The daytime sleepiness was assessed using the Epworth Sleepiness Scale (ESS). This included 8 items that explored different daily situations. Each item was coted 0 to 3, for a total score of 0 to 24. Excessive daytime sleepiness was defined by an ESS score >10 [29].

**Figure 1:**
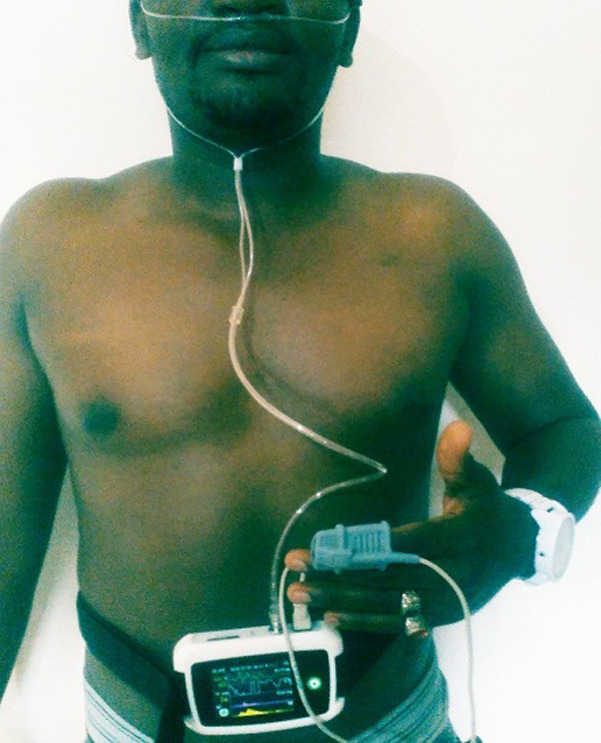
the polygraph used in the study, connected to a male adult, study on obstructive sleep apnea, Bandjoun, November 2015-April 2016

The STOPBANG score was calculated after the interview. This screening tool was developed in a pre-operative Caucasian population, and includes 8 items with “Yes” or “No” answers modality: Snoring, daytime Tiredness, Observed apnea, high blood Pressure (HBP), Body mass index (BMI) >30kg/m^2^ (obesity), Age >50 years, Neck circumference >40cm and male Gender. Each item coted “Yes” worth 1 point, and the score is the sum of points. In the original population, the STOPBANG sensitivity at threshold 3 was 93% for moderate OSAHS (apnea hypopnea index (AHI)=15-30/h) detection and 100% for severe one (AHI >30/h) [27, 28]. For each selected participant, the nocturnal RP recording was preferably done the night following his interview. This was performed using a Sleep Fairy (Qingdao Harmony International Trade Co.Ltd. Qingdao, China) branded polygraph. Measures included airflow through a nasal cannula, thoracic and abdominal movements by body plethysmography, ox hemoglobin saturation, heart rate, body position and snoring. The device is shown in Figure 1. The RPs were analyzed by two trained pulmonologists. The output included AHI value, the diagnosis of OSAHS (AHI ≥5/hr), its severity expressed as mild (AHI=5-15/hr), moderate to severe (AHI >15/hr), and oxygen desaturation features (index, mean, minimum and maximum). Participants whose recording time was < 4 hours and those with more than 25% of imperfect signal were excluded from the analysis.

**Data management and analysis:** for the main epidemiological study, the expected global sample size using Epi Info 7 was 1859, based on 2% OSAHS prevalence, 1% precision, 1.3 design effect and 10% predicted non-response rate. The number of nocturnal RPs was estimated as described above (Sampling section). We used Epidata entry 3.1 software for data collection, and IBM-SPSS Version 20 on Windows (SPSS Inc. Chicago, IL) for analysis. Categorical variables were presented as numbers and proportions, and quantitative variables as means and standard deviations (SD). We compared polygraphy and non-polygraphy groups using Chi square or Fisher tests for factors, and T Student or Mann Withney tests for continuous variables. The same tests were used to find out correlations between HR-OSAHS and other features. Power analysis was performed for the STOPBANG sensitivity, using the *pwr.p.test* function of R version 3.6.0, considering 80% sensitivity as reference. A 5% p-value level was used during the whole analysis process.

**Operational definitions:** OSAHS was defined as AHI ≥5 events per hour. Moderate to severe OSAHS (MS-OSAHS) was defined as AHI ≥15 or more. The high risk of OSAHS was defined as the presence of at least 3 “yes” responses to STOPBANG questionnaire. The predictive values (sensitivity, specificity, positive and negative predictive values) were determined by the standard equations.

**Ethic statement:** the National Ethics Committee for Human Health Research in Cameroon and the West Region’s health authorities approved the study. We further obtained a recruitment authorization from local authorities in each visited area. Each participant gave a verbal consent, after awareness of the information form read by the investigator. This verbal procedure was imposed by the working conditions: investigators had to cover great distances between houses, districts and villages; they collected data directly on computer and did not carry paper. Furthermore, some participants (illiterate and/or elder) were unable to read or fully understand written form.

## Results

**Figure 2:**
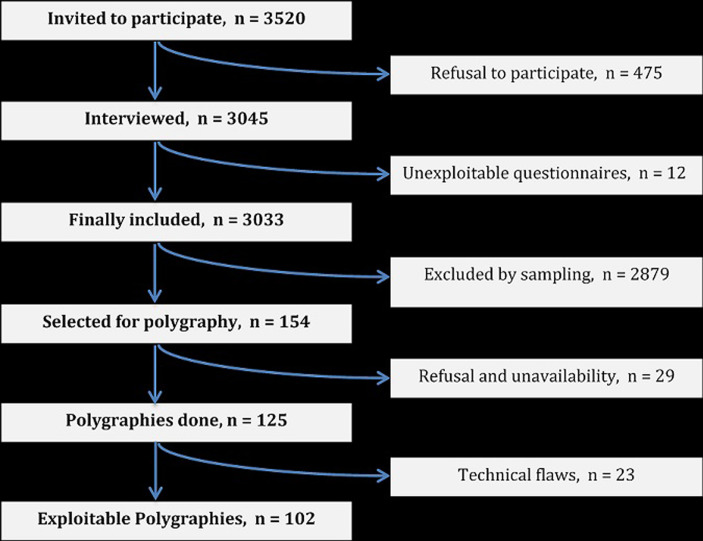
flow diagram of subject’s inclusion, study on obstructive sleep apnea, Bandjoun, November 2015-April 2016

**Recruitment of subjects:** initially, 3520 subjects were invited to participate to our study, of whom 154 were selected for polygraphs and 102 finally presented an exploitable RP, as shown on Figure 2.

**Study population:** compared with the other group, participants who benefited from a RP were significantly older (mean age 49.1±17.9 vs 41.8±18.0, P<0.001), they had a higher frequency of hypertension (11.6% vs 7.0%, p<0.001), diabetes mellitus (6.8% vs 2.0%, p=0.005) and smoking (23.5% vs 14.7%, p=0.014). They also had a higher mean BMI (29.1±6.1 vs 27.5±5.2, p=0.004). There was non-significant men predominance in the polygraph group. These characteristics are represented on Table 1.

**Table 1:** compared characteristics of subjects from polygraphy and non-polygraphy groups, Bandjoun, November 2015-April 2016

Variable	Polygraphy N = 102	No polygraphy N = 2931	p-value
Mean age, years	49.1±17.9	41.8±18.0	<0.001
Age range, years	19 - 80	19 - 96	
Male	51 (50.0)	1294 (44.1)	0.242
Mean BMI, kg/m^2^	29.1±6.1	27.5±5.2	0.001
Smokers	24 (23.5)	431 (14.7)	0.014
Medications users	30 (29.4)	527 (18.0)	0.003
High blood pressure	27 (11.6)	206 (7.0)	<0.001
Diabetes	7 (7.0)	60 (2.0)	0.005
History of stroke	1 (1.0)	11 (0.4)	0.337
Heart failure	2 (2.0)	21 (0.7)	0.180

Continuous variables are expressed in mean±standard deviation, factors are expressed in count (frequency in %)

**STOPBANG performance:** numbers and frequencies for OSAHS and MS-OSAHS according to the STOPBANG status are summarized on Table 2. The overall prevalence of OSAHS in the RP group was 40.2%, this prevalence was higher in the positive STOPBANG group (45.9%) than in the negative one (25.0%). The same trend was observed for the moderate to severe OSAHS: 18.9% vs 3.6% respectively, with 14.7% as overall prevalence. The STOPBANG sensitivity and negative predictive value (NPV) were 82.9% and 75.0% respectively for OSAHS and improved for MS-OSAHS (93.3% and 96.4% respectively). The values of specificity and positive predictive value (PPV) were quite lower (34.4% and 45.9% for OSAHS; 31.0 and 18.9 for MS-OSAHS, respectively). These data are summarized in Table 3. The power analysis for the STOPBANG sensitivity gave 19.3% for OSAHS and 97.7% for MS-OSAHS. An association was found between the STOPBANG score and some OSAHS features. Compared with the negative STOPBANG group (score ≤2), the positive one showed a greater mean AHI (9.1±10.7/hr vs 3.8±3.5/hr, p<0.001) and oxygen desaturation index (6.4±7.9/hr vs 2.6±2.1/hr, p<0.001). The ESS mean score was also greater in the positive STOPBANG group but not significantly (6.3±5.3 vs 4.5±3.5, p=0.06).

**Table 2:** frequencies of obstructive sleep apnea-hypopnea syndrome (OSAHS) and moderate to severe OSAHS (MS-OSAHS) according to the STOPBANG status, Bandjoun, November 2015-April 2016

STOPBANG	OSAHS		MS-OSAHS		Total
	Present	Absent	Present	Absent	
Positive	34 (45.9%)	40 (54.1%)	14 (18.9%)	60 (81.1%)	74 (100.0%)
Negative	7 (25.0%)	21 (75.0%)	1 (3.6%)	27 (96.4%)	28 (100.0%)
Total	41 (40.2%)	61 (59.8%)	15 (14.7%)	87 (85.3%)	102 (100.0%)

**Table 3:** predictive parameters of the STOPBANG score for OSAHS an MS-OSAHS diagnosis (n=102), Bandjoun, November 2015-April 2016

Condition	Title	Value	95% CI
OSAHS [AHI ≥ 5]	Sensitivity, %	82.9	67.9-92.8
	Specificity, %	34.4	22.7-47.7
	Positive predictive value, %	45.9	34.3-57.9
	Negative predictive value, %	75.0	55.1-89.3
MS-OSAHS [AHI ≥ 15]	Sensitivity, %	93.3	68.0-99.0
	Specificity, %	31.4	21.5-41.9
	Positive predictive value, %	18.9	10.7-29.7
	Negative predictive value, %	96.4	81.6-99.9

## Discussion

**Principal findings:** some characteristics (Age, smoking status, BMI, hypertension, and diabetes) of the 102 polygraph group’s subjects differed from those of the global sample surveyed. The prevalence of OSAHS in the RP group was 40.2%. Concerning STOPBANG diagnostic performance for global and moderate to severe OSAHS respectively, sensitivity was of 82.9 and 93.3%, specificity was 34.4 and 31.0%, PPV was 45.9 and 18.9% and NPV was 75.0 and 96.4%. The STOPBANG-based HR-OSAHS was associated with AHI, oxygen desaturation index and ESS.

**External validity:** the higher age, BMI and hypertension rate in the VPG group could lead to an overestimation of diagnostic performance, since those 3 features are part of the STOPBANG questionnaire. However, in the main STOPBANG validation study, the characteristics of patients in polysomnography group (177 patients) were even more at risk than those of our patients: average BMI =30±7kg/m^2^, average age =56±13 years, sex ratio H/F=1.01 [27]. Our OSAHS prevalence appeared to be slightly higher than the one estimated by Benjafield (36.7%) in his recent review, concerning Cameroon as a whole [5]. However, it remained within the sub-region estimated range [5]. Our STOPBANG sensitivity for the diagnosis of global OSHAS (82.9%) was close to the 83.6% found during the development of this score, but we found it to be a weaker power test. This was probably due to our smaller sample size. The NPV, which is the second most important feature of a screening test, was better in our study (75% vs. 60.8%), while specificity and PPV were worse (34.4% vs 56.4% and 45.9% vs 81% respectively). These last ones are less detrimental for a screening tool. The STOPBANG performance improved for MS-OSAHS screening. Compared with the original study, the sensitivity and NPV trends were maintained (93.3% vs 92.9% and 96.4% vs 90.2% respectively), while the difference was lesser for the other two performances (Specificity 31% vs 43% and PPV 18.9% vs 51.6%). We found no STOPBANG validation study conducted in SSA, to compare our results. In 2013, Boynton and coauthors assessed STOPBANG diagnostic performance among sleep clinic patients in two American university hospitals. They found a sensitivity of 82, 93, and 97% for mild, moderate and severe OSAHS respectively; and NPV of 44, 87, and 96% [30]. Those results are close to ours, despite the difference in study populations, suggesting a good stability of the STOPBANG questionnaire regardless of settings.

**Internal validity:** our study presented some limitations. The West Region is known to have a high-fat diet, leading to suspected higher overweight and obesity prevalence. This population is therefore probably not representative of the country. Our resulting RP sample was therefore probably even less representative, which makes the extrapolation of our results tricky. The number of RPs performed was limited and may have contributed to the disruption of our results. The high refusal and withdrawal/unavailability rates (18.8%) were one of the causes of this limited number of polygraphies and could partly explain the differences between RP group and overall sample. This could occur through a self-selection phenomenon: the more symptomatic, older, smoker and obese subjects could be more health-concerned and accept more willingly to undergo screening tests. The consequence of this self-selection may have overestimated the sensitivity of our screening tool. The high frequency of non-exploitable RP records (18.4%) may also have skewed our results. This could be explained by a progressive experience acquirement for the investigators, accidental sensors removal during sleep and unexplained interruptions of signal capture. However, the randomness of these technical flaws should minimize their effect on the STOPBANG diagnostic performance. The calculated power of STOPBANG sensitivity was low for OSAHS and high for MS-OSAHS, probably due to a larger clinical gap (between measured sensitivity and reference) for the latter. The positive association found with AHI and oxygen desaturation index was an additional argument in favor of STOPBANG effectiveness in predicting OSAHS. Strengths of our study included the random sampling and the number of participants enrolled for the overall study. In our knowledge, this was the first community study using a nocturnal polygraphy in Cameroon.

**Key message:** despite the limitations, our STOPBANG score performance was close to the original one, especially in MS-OSAHS detection, which is the main target for treatment. However, these limitations should lead to caution and encourage additional studies with larger and more representative populations.

## Conclusion

In this sub-Saharan African community-based validation study, the STOPBANG performance for OSAHS screening was comparable to that previously described in Caucasian populations. Physicians in SSA could therefore be encouraged to use this simple, costless, and efficient tool, for the OSAHS screening on selected patients. Obesity and other cardiovascular risk factors frequently associated with OSAHS are increasing in SSA, and the affordability of sleep disorders diagnosis devices is still very low. However, there is still need for diagnostic devices availability, to conduct reliable polygraphy-based community and clinical studies.

### What is known about this topic

- Studies on obstructive sleep apnea-hypopnea syndrome (OSAHS) are scarce in sub-Saharan Africa;
- The effectiveness of the STOPBANG score in screening OSAHS has been established in western population.

### What this study adds

- The prevalence of OSAHS was high in this sub-urban Cameroonian population but was consistent with national and sub-regional estimation;
- The STOPBANG score for OSAHS screening in this SSA population, seems to be as effective as in the Western settings.
